# MiR-191 as a Key Molecule in Aneurysmal Aortic Remodeling

**DOI:** 10.3390/biom11111611

**Published:** 2021-10-30

**Authors:** Sabina Lichołai, Dorota Studzińska, Hanna Plutecka, Tomasz Gubała, Wojciech Szczeklik, Marek Sanak

**Affiliations:** 1 Division of Molecular Biology and Clinical Genetics, Faculty of Medicine, Jagiellonian University Medical College, Skawinska 8, 31-066 Kraków, Poland; hanka.plutecka@uj.edu.pl (H.P.); marek.sanak@uj.edu.pl (M.S.); 2 Department of Intensive Care and Perioperative Medicine, Faculty of Medicine, Jagiellonian University Medical College, Wrocławska 1/3, 30-901 Kraków, Poland; d.studzinska@uj.edu.pl (D.S.); wojciech.szczeklik@uj.edu.pl (W.S.); 3 Sano Centre for Computational Medicine, Czarnowiejska 36, 30-054 Kraków, Poland; t.gubala@sano.science

**Keywords:** abdominal aortic aneurysms, microRNA, endothelial dysfunction

## Abstract

Abdominal aortic aneurysms (AAA) are a complex disease with an unclear pathomechanism. A positive family history is emphasized as a significant risk factor, and a nonspecific model of inheritance suggests participation of epigenetic regulation in the pathogenesis of this disease. Past studies have implicated microRNAs in the development of AAA; therefore in this project, we measured miR-191 levels in AAA patients and compared them with a control group. We found that miR-191 levels were significantly elevated in aneurysmal patients, although this did not correlate with the available clinical data. We then developed an in vitro model where, using cells with an endothelial phenotype, we determined the effect of miR-191 on the transcriptome using RNA sequencing. Subsequent pathway analysis established that some of the perturbations mediated by miR-191 can be explained by several processes which have long been observed and described in literature as accompanying the development of abdominal aortic aneurysms.

## 1. Introduction

Abdominal aortic aneurysm (AAA) is defined as local dilatation of the main artery, exceeding its normal diameter by 50% [1]. The pathogenesis of the disease is still unclear, but some risk factors has been established. Tobacco smoking, hypertension, male sex and advanced age increase the risk of developing the disease [1]. On a molecular level, pathological processes involved in the formation of AAA include upregulation of proteolytic pathways, apoptosis of structural cells, oxidative stress, inflammation, and loss of the arterial wall matrix [2]. Although AAA presents as a focal lesion, some evidence suggests that it can be regarded as a systemic disease and that the entire vascular system is impaired in patients with AAA, although specific molecular mechanisms remain unknown [3]. A positive family history is also a risk factor, even though its genetic background is not fully understood [4]. Moreover, there is no consistent model of inheritance in AAA [5]. All this points to possible involvement of epigenetic factors in the pathogenesis of this disease.

The concept of epigenetics suggests regulation of gene expression by means other than direct changes in the DNA sequence [6]. The human genome contains noncoding RNA (ncRNA) genes that are not translated into proteins but contribute to the regulatory network [7]. MicroRNAs (miRNAs or miRs) are endogenous noncoding RNAs which play an important role in posttranscriptional gene regulation [8]. The action of microRNAs is redundant by nature. A single microRNA molecule can regulate the expression of numerous genes by binding (in a completely or partially complementary manner) to its target site within their respective sequences. At the same time, a single gene can be simultaneously regulated by multiple microRNA molecules. Therefore, it is thought that the synergistic effect of these molecules is mainly to modulate the expression levels of multiple genes at the transcriptome level. Nevertheless, they have been shown to participate in many biological processes, including cell cycle regulation, apoptosis, and angiogenesis. In addition, they are known to be involved in the pathogenesis of many human diseases, including cancer [9]. The role of microRNA in the pathogenesis of AAA has not been fully explained. In our previous study we described differences in microRNA levels between the aneurysm and normal aortic wall [10]. One of the molecules with the highest observed difference in expression levels was miR-191.

MiR-191 is encoded by the *MIR191* gene located on chromosome 3. The exact function of this microRNA remains unclear, but it has been proposed to be an anti-proliferative and anti-replicative microRNA associated with aging in primary human keratinocytes [11]. MiR-191 overexpression was sufficient to induce senescence of these cells. It has also been shown to target 3’UTRs of SATB1 and CDK6 transcripts involved in neurosensory senescence and cell cycle arrest. Therefore, the aim of the present study was to attempt to relate the effects of microRNAs upon endothelial cells essential for aneurysm formation to the previously observed characteristic abnormalities that have long been described in the context of aortic aneurysm formation. Thus, in the current study we evaluated the level of circulating miR-191 in a group of AAA patients, and compared it to control individuals with the same risk factors. Then, using an in vitro cellular model, we investigated the effects of miR-191 on gene expression at the transcriptome level, and performed pathway analysis of differentially expressed genes. 

## 2. Materials and Methods

### 2.1. Patients and Controls

The study group was composed of 205 patients with AAA, whose clinical data and serum samples were available at the local biobank of the Division of Molecular Biology and Clinical Genetics, Department of Internal Medicine, Jagiellonian University Medical College. Patients were recruited from those admitted for surgical stent graft implantation. Informed consent to participate was collected from each patient, and tissue and serum samples were then preserved at a local biobank. The control group included 180 patients with peripheral arterial occlusive disease, matched with AAA patients by demographic characteristic using the propensity score (PS) method. Baseline variables for calculating PS included age, sex, BMI, hypertension, atherosclerosis, coronary artery disease and tobacco smoking. The demographic characteristics of patients and controls are presented in Table 1 and Table 2. The study protocol was approved by the Bioethics Committee of the Regional Medical Chamber in Kraków (12/KBL/OIL/2010) and the Bioethics Committee of the Jagiellonian University Medical College (KBET/232/B/2013).

### 2.2. Assessment of Circulating miR-191 Concentration

RNA was isolated from the frozen serum as a small RNA fraction using a commercially available microcolumn-based silica kit (A&A Biotechnology, Gdansk, Poland) with spike-in RNA (dme-miR-7) and reverse transcribed using the TaqMan Advanced miRNA cDNA Synthesis Kit (ThermoFisher, Waltham, MA, USA) according to the manufacturer’s protocol. The specific cDNA was quantified by real-time PCR amplification using commercially available TaqMan specific probes for miR-191 and miR-16 using the 7900HT Fast Real-Time PCR System. Raw amplification data was preprocessed using the SDS 2.4 software (ThermoFisher, Waltham, MA, USA) and exported for further analysis. The fold change of miR expression was calculated using the 2^−∆∆CT^ method and the threshold cycle was ascertained using the maximum of second derivative method. All sample data was corrected for extraction and reverse transcription efficiency using spike-in miR (dme-miR-7), followed by amplification adjusted to the miR-16 signal as endogenous control. The Man-Whitney rank test was used to determine significance, and the level of significance was set at 0.05. A fold change of more than 2 or less than 0.5 was regarded as significant.

### 2.3. Cell Cultures

iCell Endothelial Cells (Cellular Dynamics, Madison, WI, USA), purified human endothelial cells derived from induced pluripotent stem cells, were cultured in Vasculite Maintenance Medium (Cellular Dynamics) according to the manufacturer’s protocol. For all experiments, cells were used between passage 2 and 5 and at 70–85% confluence. 

### 2.4. Transfection of Cultured Cells 

Transfection with miR-191 mimic was performed using HiPerfect Transfection Reagent (Qiagen), according to the manufacturer’s protocol. Briefly, cells were seeded in a 24-well plate the day before transfection and were incubated under normal growth conditions until 70–85% confluence was reached. The 100 pmol of miR-191 mimic was mixed with HiPerfect Transfection Reagent and culture medium, and then incubated for 10 min to enable formation of the transfection complex. The transfection media was then incubated with endothelial cells under normal growth conditions for 12 h. All experiments were performed in triplicate. As a negative control, mock transfection with empty complexes (without miRNA mimic) was performed to measure baseline expression levels. 

### 2.5. RNA Isolation from Endothelial Cells 

Total RNA was isolated using a standard RNA purification method on minicolumns according to the manufacturer’s protocol (TotalRNA Mini, A&A Biotechnology, Gdansk, Poland). The concentration and purity of the RNA eluent were measured by spectrophotometry (NanoDrop Spectrophotometer 2000, NanoDrop Technologies, Wilmington, DE, USA). Quality and integrity of RNA was assessed with Bioanalyzer (PicoRNA Chip, Agilent Technologies, Santa Clara, CA, USA). 

### 2.6. Library Construction and RNA-Sequencing

For library construction, 100 ng of total RNA per sample was used as starting material, with only high-quality RNA samples (RIN ≥ 9) used. This step was performed according to the standard protocol from Illumina allowing for ribosomal depletion. RNA sequencing was performed with Illumina reagents and 2500 HiSeq platform (Illumina) at a genomic core facility. The average sequence reads equalled 20 million in 2 × 100 bp paired-end fragments for each sample.

### 2.7. Bioinformatic Analysis of RNA-Seq Reads

Raw base calls files (.bcl files) were converted to .fastq using the bcl2fastq converter. Alignment of paired ends was performed using the STAR package (2.7.0a), which aligned RNA-seq reads to the human genome in a mode which provided correction for spliced junctions. The Salmon tool (1.3.0) was used to assess the expression level for each gene, and protein-coding transcripts were then selected. Differential analysis compared transcripts in transfected versus mock transfected cells regarding potential protein coding mRNAs and was performed using the deseq2 R package (1.6.3). This step was carried out to screen for differentially expressed gene (DEG) transcripts based on the human reference transcriptome, detecting DEGs of known function. 

### 2.8. Pathway Analysis

Genes whose relative expression exceeded 1.4 were subjected to pathway analysis in order to explore biological processes potentially affected by miR-191. This analysis was performed using the Reactome database, with visualizations using the ReactomePA package (1.4.1) for R.

## 3. Results

### 3.1. Comparison of Circulating miR-191 between Patients and Controls

**Figure 1:**
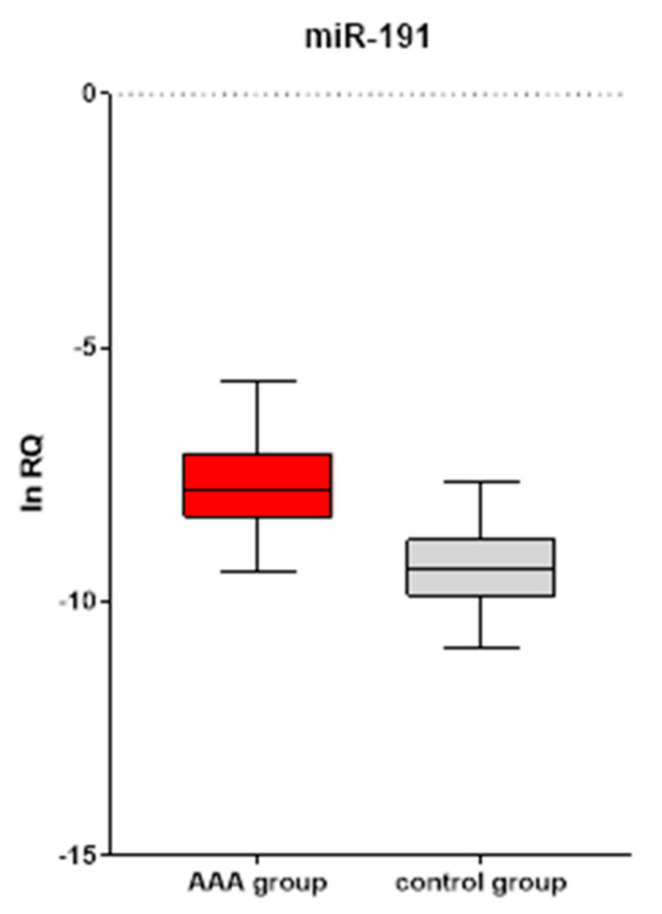
Difference in serum levels of miR-191 between patients and the control group. Values are presented as a natural logarithm of relative quantification.

The study involved 205 patients with AAA and 180 controls. The level of circulating miR-191 was assessed using specific TaqMan Assays in total RNA isolated from serum samples. The serum levels of miR-191 were higher in patients with AAA compared to the control group (*p* < 0.05). Results are summarized in Figure 1. No significant correlation was detected between miR-191 levels and age, time to disease progression, or individual AAA risk factors.

### 3.2. Differences in Gene Expression Profiles of Endothelial Cells after Transfection with miR-191 

**Figure 2:**
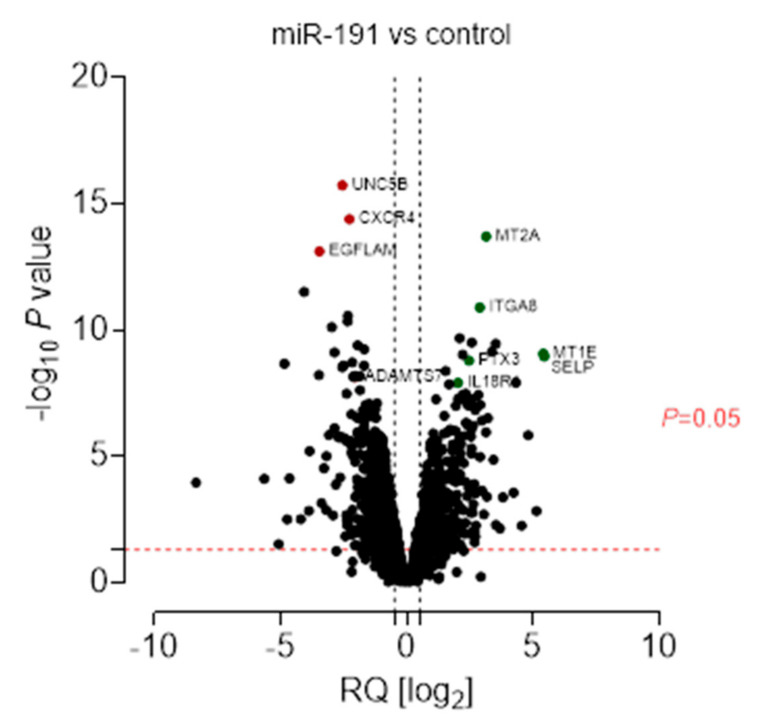
Altered expression profile of protein-coding genes in endothelial cells induced by miR-191. Results summarized as a volcano plot.

To investigate the effect of miR-191 overexpression on the global gene expression pattern, we used an in vitro model of endothelial cells and transfected them with the miR-191 mimic molecule. Total RNA from the cells was isolated 12 h after transfection and deep RNA sequencing was performed. cDNA libraries from miR-191 and mock-transfected cells were sequenced after ribosomal depletion. The results indicated that miR-191 transfection induced significant changes in the expression of 1492 protein coding genes (log fold change ≥ |1.4|). Results are presented as a volcano plot in Figure 2, while a list of 50 most up- and downregulated genes is presented in Table S1 (Supplementary Materials). 

### 3.3. Comparison between Potential Targets of miR-191 and Differentially Expressed Genes

A single miRNA can potentially target hundreds of genes. To explore the possibility that global changes in the expression pattern observed after miR-191 transfection were the result of direct targeting, we first obtained a list of potential miR-191 targets predicted by three major target prediction algorithms (miRanda, TargetScan, and PicTar). We then compared the aggregated list of targets with the list of significantly downregulated genes. Results indicated that 17 downregulated genes which were expressed differently in the wake of miR-191 transfection possessed target sequence motifs for this miRNA.

### 3.4. Pathway Analysis

**Figure 3:**
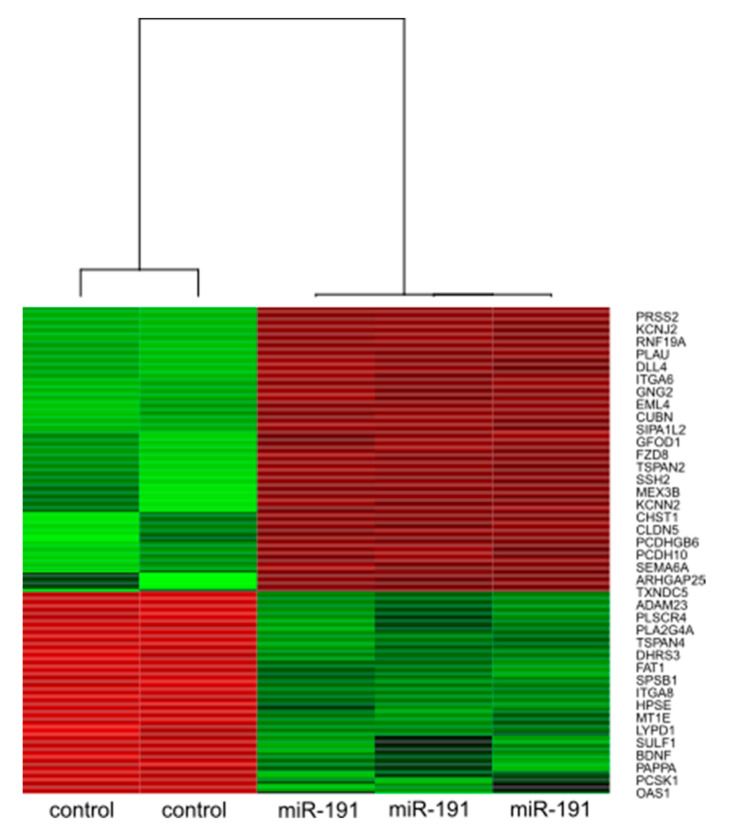
Results of cluster analysis summarized as a heatmap.

The differentially expressed genes were subjected to pathway analysis to identify biological processes potentially modulated by miR-191. First, cluster analysis was performed using the Euclidean metric and a heat map was generated (Figure 3). The Reactome database was then used to identify biological pathways potentially affected by miR-191. The results of our functional pathway analysis indicated that genes differentially expressed after miR-191 transfection were significantly overrepresented in at least 27 functional pathways. The most significantly overrepresented pathways are listed in Table S3 (Supplementary Materials) and include cell adhesion, metal-binding metallothioneins, immune activation and interleukin signalling. DNA methylation and extracellular matrix metabolism also appear to be impaired. 

## 4. Discussion

Aortic aneurysm is defined as a permanent, localized dilatation of the aorta encompassing all three layers of the vessel wall and exceeding its normal diameter by 50% [12]. The pathomechanism underlying aortic aneurysm involves permanent weakening of the aortic wall, which, if untreated, leads to progressive dilatation and eventual rupture [1]. Surgery is the only efficient treatment for patients with aneurysms, however, it is still associated with a high risk of serious complications, including stroke, cardiovascular events and death [13]. On the other hand, no effective non-surgical treatment is currently available, mostly due to the unclear pathogenesis of the disease, and there is strong evidence implicating microRNAs in the process of pathological aneurysmal formation. 

Traditionally, a distinction is made between aortic aneurysms which are manifestations of congenital syndromes, and those which occur as a sporadic disease. In the case of the former, Marfan syndrome or Loeys-Dietz syndrome (among others) are often diagnosed [14]. Studies using transgenic mice with fibrillin gene mutation modelling Marfan syndrome have demonstrated the critical role of elevated TGF-β signalling in promoting abnormal vascular remodelling, dilation, and enlargement of aneurysms [15]. These findings were confirmed by studies demonstrating increased TGF-β mediated signalling in patients with Marfan syndrome and by identification of gain-of-function mutations within the *TGFBR1* and *TGBR2* genes in patients with Loyes-Dietz syndrome [16,17]. In addition, non-syndromic forms of aortic aneurysms also exhibit increased vascular TGF-β expression and SMAD-2 phosphorylation [18]. Overactive TGF-β signalling appears to be particularly important when considering the potential link between miR-191 and the pathogenesis of aortic aneurysms. MiR-191 is an important regulator, affecting endothelial cell function, including at the stage of differentiation from stem cells, interacting with the same TGF-β pathway and directly targeting SMAD-2, both of which are important in disease pathogenesis [19].

In the first part of our study, we verified whether there was a difference in the level of circulating microRNA between patients and controls. Indeed, miR-191 levels were significantly higher among patients with AAA as compared to the controls. At the same time, statistical analysis revealed no association between the level of this miRNA and demographic characteristics. Our results are in line with those of Tenorio et al. [20]. Although they used whole blood as material for RNA isolation, and their cohort was much smaller than ours (30 vs. 180 patients), they reported elevated miR-191 levels in patients with AAA. Interestingly, at a 6-month follow-up, these levels were found to have decreased significantly following invasive treatment. Moreover, similarly to our results, they found no correlation between miR-191 levels and disease severity. Similar conclusions were reached by a group from Ninjang University (Zhang et al.) who performed a screening test on 10 patients and 10 controls [21]. Of the numerous molecules measured, miR-191 turned out to exhibit one of the most altered expression profiles. Although their study was conducted on a population with a different ethnic background than ours, the results were consistent with ours. We obtained confirmation on a much larger group, which strongly supports potential implication of miR-191 in the pathogenesis of the disease; thus, we decided to investigate the molecular consequences of miR-191 overexpression in an in vitro model that would allow for a more thorough analysis of the transcriptome.

Circulating microRNA can affect vascular endothelial cells, which cushion the entire circulatory system and play a pivotal role in maintaining homeostasis [22]. For instance, the endothelium regulates vascular tone, platelet activity, leucocyte adhesion and angiogenesis [23]. The role of the endothelium is exerted through the production of nitric oxide and other regulatory factors, as well as through the presence of membrane-bound receptors for numerous molecules [23]. On the other hand, endothelial dysfunction plays a central role in the pathogenesis of a broad spectrum of human diseases, including peripheral vascular diseases, stroke, heart diseases, diabetes, aortic aneurysms and inflammatory vascular diseases [22]. However, it is not clear whether dysregulation of endothelial cell function is the cause of the disease or its result. Studies accumulated in recent years indicate that disturbances in epigenetic regulation of biological processes could contribute to the dysfunction of the endothelium [24]. Among other factors, endothelial dysfunction is considered to be one of the causes of aneurysms. We hypothesized that circulating miR-191, whose levels are elevated in patients with AAA, may affect available endothelial cells and lead to deleterious changes ultimately associated with disease progression. The principal action of miRNAs occurs in the cytoplasm of the cell, where miRNAs specifically target mRNAs [8]. Gene expression is regulated by miRNAs and is an important aspect of the field of epigenetics, as this modulation is achieved without altering the sequence of nucleic acids [7]. The activity of miRNAs in regulating gene expression and cellular physiology appears to be widespread [25]. The action of miRNAs is pleiotropic in that a single miRNA molecule has hundreds of different targets for mRNAs, and one mRNA can be targeted by multiple miRNAs [26]. The action of miRNAs targeting the same mRNA is either synergistic or antagonistic. Moreover, most algorithms produce widely divergent predictions of partially complementary miRNA target sites with a substantial number of false positives and false negatives that are difficult to verify in silico [9]. Consequently, targets identified in silico should be subjected to experimental validation. Thus, in our study, besides bioinformatic analysis, we exposedendothelial cells to miR-191, as occurs in an aneurysmal aorta. We then performed a comprehensive analysis of the transcriptome, focusing on its coding fraction, to seek characteristic endothelial phenotypes. Our results indicate that changes induced by miR-191 may explain some of the molecular abnormalities observed in AAA.

One of the most characteristic elements whose involvement in the pathogenesis of aneurysms is highly emphasized is excessive activation of extracellular matrix metalloproteinases [27]. Pathway analysis indicates that metallothioneins are overexpressed in our in vitro model. Metallothionein 1E (MT1E) belongs to a family of low molecular weight metalloproteinases that exhibit high affinity for heavy metal ions such as zinc, cadmium, copper, mercury and platinum [28]. This implies, among others, that MT1E may be involved in induction of matrix metalloproteinases, which is observed during aneurysm formation. Furthermore, miR-191 has been reported to accompany tumor progression and metastasis, and has also been described as associated with overexpression of matrix metalloproteinases. On the other hand, MT1E participates in fundamental cellular processes, such as cell proliferation and apoptosis [29]. Thus, overexpression of miR-191, which leads to upregulated of MT1E, may partially explain the phenomena observed during aneurysmal formation and progression.

Another widely highlighted component of aneurysm pathogenesis is the loss of extracellular matrix (ECM) integrity [2]. The extracellular matrix is extremally important for almost all aspects of vascular biology. It provides support for the vascular endothelium, primarily through adhesive interactions with integrins on its surface [30]. In addition, adhesion of the ECM is essential for the proliferation, migration, morphogenesis, survival, and, ultimately, stabilization of the blood vessels, all of which are critical for neovascularization [30]. All of these processes appear to be impaired in patients with AAA. Our functional analysis revealed that pathways associated with extracellular matrix remodeling are also affected by miR-191 overexpression.

In addition, studies published in recent years suggest a potential contribution of immune system dysfunction to the pathogenesis of AAA. Within the aneurysmal aorta wall, infiltration of T lymphocytes is observed, mainly of the CD4+ phenotype, accompanied by CD8+ [31]. Possible involvement of relatively recently discovered lymphocytes such as Th17 or Treg is also underlined [32], as is the potential contribution of neutrophils. Therefore, overrepresentation of genes related to the “Immune Regulation” pathway seems to support this hypothesis. These results are in line with the findings of Lykken et al. In their study, they identified miR-191 as a key regulator of naive, memory and regulatory T cell homeostasis. Downregulation of miR-191 resulted in preferential loss of peripheral CD4+ regulatory T cells, as well as naive T cells and memory CD8+ cells. These preferential losses are due to reduced survival following attenuated cytokine signalling and STAT5 activation [33]. Moreover, the overrepresentation of adhesion molecules may cause facilitated adherence, resulting in infiltration of immune system cells, which also explains the observed features. Eventually, specific signalling pathways mediated by Il-35, Il-27, Il-18, and Il-33 were identified as disrupted in our analyses. These abnormalities are in agreement with the effects of interleukins observed in other studies. Among others, Il-35 induces Il-32 in smooth muscle cells of the vessel wall [34]. Its levels were shown to be elevated in AAA samples, with particular emphasis on endothelial cells, smooth muscle cells as well as immune cells [35]. In turn, Il-27 via its receptor Il-27R significantly accelerates aneurysm formation in a mouse model of the disease [36]. Il-18, on the other hand, has been linked to AAA formation through MMP activation or macrophage recruitment [37]

The main limitation of the present study is the lack of direct proof for miR-191 contribution to aneurysm formation. In an animal model overexpressing miR-191, prospective observation from the organogenesis of the aorta to its dilatation and eventual rupture would provide the required evidence. Therefore, the present work cannot be taken as a confirmation of a causal relationship between miR-191 overexpression and abdominal aortic aneurysm formation. In the present study, we began by observing that miR-191 levels were significantly elevated in patients with abdominal aortic aneurysms, and then we demonstrated, using an in vitro model, the consequences of elevated levels of one particular microRNA molecule for the endothelial cell transcriptome. Subsequent pathway analysis enabled us to suggest which mechanisms may have been disrupted. Comparison with known mechanisms of AAA pathogenesis led us to confirm those which had already been suggested, as well as to propose a novel one, based on the immunoregulatory function of miR-191. Nevertheless, future experiments using animal models of AAA will be necessary to fully validate the results presented above.

## 5. Conclusions

To summarize, in this study we aimed to elucidate the involvement of miR-191 in AAA. First, we determined the levels of miR-191 in AAA patients compared with controls, and demonstrated significant overexpression of the molecule in the disease. Then, using an in vitro model, we reproduced the effects of miR-191 on vascular endothelial cells and quantified the changes at the transcriptome level. As a result, we confirmed some of the molecular processes previously described as components of AAA pathogenesis, which can be explained by the impact of miR-191 on endothelial cells. Though this study design cannot prove that miR-191 is the cause of aortic aneurysms, it can definitely be regarded as a companion molecule in the developing pathology. With appropriate measurement techniques coupled with established reference values, it could, in the future, be used as a biomarker of the disease.

## Figures and Tables

**Table 1:** Clinical characteristics of the study and control groups.

	AAA [*n* = 205]			Control Group [*n* = 180]			*p* Value
Age [years, mean ± SD]	68.98	±	7.85	67.57	±	7.32	0.3212
Male sex [*n*, %]	170		82.93%	160		80.00%	0.4484
Hypertension [*n*, %]	158		77.07%	156		78.00%	0.8232
Coronary artery disease [*n*, %]	96		46.83%	97		48.50%	0.7367
Cardiac failure [*n*, %]	21		10.24%	32		16.00%	0.8593
Aortic stenosis [*n*, %]	7		3.41%	6		3.00%	0.4812
Deep vein thrombosis [*n*, %]	6		2.93%	7		3.50%	0.7435
Pulmonary embolism [*n*, %]	2		0.98%	3		1.50%	0.6327
Stroke [*n*, %]	9		4.39%	16		8.00%	0.1312
TIA [*n*, %]	2		0.98%	2		1.00%	0.9747
Obstructive sleep apnoea [*n*, %]	7		3.41%	6		3.00%	0.8129
Gastric ulcer [*n*, %]	4		1.95%	2		1.00%	0.4280
Analgetic therapy [*n*, %]	11		5.37%	15		7.50%	0.3810
Nicotine [*n*, %]	130		63.41%	124		62.00%	0.7680
Kidney failure [*n*, %]	3		1.46%	2		1.00%	0.6730

**Table 2:** Morphological description of aneurysms in the study group.

	AAA [*n* = 205]		
Age [years, mean ± SD]	68.98	±	7.85
Age at the moment of diagnosis [years, mean ± SD]	66.67	±	8.45
Diameter of aneurysm [cm, mean ± SD]	5.85	±	1.43
Length of aneurysm [cm, mean ± SD]	11.38	±	3.99
Dissecting aneurysm [*n*, %]	0		0.00%
Ruptured aneurysm [*n*, %]	6		2.93%
Symptomatic aneurysm [*n*, %]	48		23.41%

## Data Availability

The data that supports the findings of this study is available from the corresponding author, S.L. upon reasonable request.

## Supplementary Materials

The following are available online at www.mdpi.com/article/10.3390/biom11111611/s1. Table S1: List of 50 most upregulated genes induced by miR-191 in endothelial cells; Table S2: List of 50 most downregulated genes after stimulation with miR-191 in endothelial cells; Table S3: List of the most perturbed pathways according to the Reactome database.
