# Supplementary material for: MiR-191 as a Key Molecule in Aneurysmal Aortic Remodeling
Source: Biomolecules. 2021 Oct 30;11(11):1611. doi: 10.3390/biom11111611 (PMC8615628; doi:10.3390/biom11111611)
Supplement: Supplementary file 1 [file biomolecules-11-01611-s001.zip › biomolecules-1364731-supplementary.pdf]

Supplementary materials.

Table S1. List of 50 most up-regulated genes induced by miR-191 in endothelial cells.

Gene name	Gene description	log2FoldChange	padj
**SELP**	selectin P	5.432	0.000
**MT1E**	metallothionein 1E	5.396	0.000
**NSG1**	Neuron-specific protein family member 1	5.130	0.000
**MT1X**	metallothionein 1X	4.802	0.000
**SDK2**	sidekick cell adhesion molecule 2	4.542	0.000
**POSTN**	periostin	4.309	0.000
**CRLF2**	cytokine receptor-like factor 2	4.220	0.000
**ALDH1A1**	aldehyde dehydrogenase 1 family member A1	3.802	0.000
**PTPRQ**	protein tyrosine phosphatase, receptor type Q	3.677	0.001
**CXCL10**	C-X-C motif chemokine ligand 10	3.521	0.002
**ART4**	ADP-ribosyltransferase 4 (Dombrock blood group)	3.508	0.000
**OAS1**	2'-5'-oligoadenylate synthetase 1	3.424	0.000
**IL1RL1**	interleukin 1 receptor like 1	3.360	0.000
**PCSK1**	proprotein convertase subtilisin/kexin type 1	3.199	0.000
**C16orf71**	chromosome 16 open reading frame 71	3.162	0.000
**MT2A**	metallothionein 2A	3.133	0.000
**CXCL12**	C-X-C motif chemokine ligand 12	3.122	0.000
**DIO2**	iodothyronine deiodinase 2	3.042	0.000
**ASS1**	argininosuccinate synthase 1	2.969	0.000
**BMP4**	bone morphogenetic protein 4	2.952	0.000
**DHRS3**	dehydrogenase/reductase 3	2.904	0.000
**TMEM88**	transmembrane protein 88	2.895	0.000
**ITGA8**	integrin subunit alpha 8	2.888	0.000
**CDH6**	cadherin 6	2.825	0.000
**CCL7**	C-C motif chemokine ligand 7	2.728	0.002
**CAMK2N1**	calcium/calmodulin dependent protein kinase II inhibitor 1	2.727	0.000
**BEX1**	brain expressed X-linked 1	2.713	0.000
**IL33**	interleukin 33	2.704	0.000
**MYRIP**	myosin VIIA and Rab interacting protein	2.700	0.015
**RAB11FIP4**	RAB11 family interacting protein 4	2.688	0.010
**FGF9**	fibroblast growth factor 9	2.678	0.026
**COL23A1**	collagen type XXIII alpha 1 chain	2.665	0.000
**UNC5D**	unc-5 netrin receptor D	2.655	0.000
**UNC13A**	unc-13 homolog A	2.626	0.000
**TINAGL1**	tubulointerstitial nephritis antigen like 1	2.614	0.000
**MAL2**	mal, T-cell differentiation protein 2 (gene/pseudogene)	2.597	0.000
**BGN**	biglycan	2.567	0.000
**SULF1**	sulfatase 1	2.566	0.000

**HAPLN3**	hyaluronan and proteoglycan link protein 3	2.564	0.000
**SPOCK1**	SPARC/osteonectin, cwcv and kazal like domains proteoglycan 1	2.557	0.000
**PDE7B**	phosphodiesterase 7B	2.552	0.000
**MINOS1-NBL1**	MINOS1-NBL1 readthrough	2.548	0.011
**PLSCR4**	phospholipid scramblase 4	2.535	0.000
**NGFR**	nerve growth factor receptor	2.533	0.000
**ANKRD35**	ankyrin repeat domain 35	2.519	0.000
**ABC7-42404400C24.1**	NA	2.493	0.008
**PTX3**	pentraxin 3	2.452	0.000
**CA12**	carbonic anhydrase 12	2.440	0.000
**GRIA3**	glutamate ionotropic receptor AMPA type subunit 3	2.410	0.000
**SELL**	selectin L	2.387	0.000

Table S2. List of 50 most down-regulated genes after stimulation with miR-191 in endothelial cells.

Gene name	Gene description	log2FoldChange	padj
**MXI1**	MAX interactor 1, dimerization protein	-2.075	0.033
**HLX**	H2.0 like homeobox	-2.135	0.000
**PRDM1**	PR/SET domain 1	-2.158	0.000
**AC007040.11**	NA	-2.162	0.005
**GPR68**	G protein-coupled receptor 68	-2.168	0.000
**PCDH12**	protocadherin 12	-2.172	0.000
**KCNQ3**	potassium voltage-gated channel subfamily Q member 3	-2.179	0.003
**AXIN2**	axin 2	-2.196	0.000
**SGIP1**	SH3 domain GRB2 like endophilin interacting protein 1	-2.206	0.000
**AFAP1L2**	actin filament associated protein 1 like 2	-2.207	0.000
**ADM**	adrenomedullin	-2.226	0.007
**CXCR4**	C-X-C motif chemokine receptor 4	-2.285	0.000
**RCAN2**	regulator of calcineurin 2	-2.286	0.000
**ARC**	activity regulated cytoskeleton associated protein	-2.318	0.001
**NID2**	nidogen 2	-2.340	0.000
**GPRC5B**	G protein-coupled receptor class C group 5 member B	-2.355	0.000
**CAMSAP3**	calmodulin regulated spectrin associated protein family member 3	-2.368	0.000
**C6orf141**	chromosome 6 open reading frame 141	-2.381	0.000
**CHST1**	carbohydrate sulfotransferase 1	-2.395	0.000
**VLDLR**	very low density lipoprotein receptor	-2.402	0.000
**CMPK2**	cytidine/uridine monophosphate kinase 2	-2.423	0.000
**ANKRD18A**	ankyrin repeat domain 18A	-2.445	0.015

**TSPAN2**	tetraspanin 2	-2.511	0.000
**PGF**	placental growth factor	-2.512	0.000
**PIK3CG**	phosphatidylinositol-4,5-bisphosphate 3-kinase catalytic subunit gamma	-2.559	0.000
**UNC5B**	unc-5 netrin receptor B	-2.567	0.000
**ITGA9**	integrin subunit alpha 9	-2.652	0.000
**FER1L6**	fer-1 like family member 6	-2.689	0.000
**PRND**	prion protein 2 (dublet)	-2.699	0.000
**TNFSF11**	tumor necrosis factor superfamily member 11	-2.821	0.000
**COL5A3**	collagen type V alpha 3 chain	-2.874	0.000
**SAT1**	spermidine/spermine N1-acetyltransferase 1	-2.876	0.000
**CPA3**	carboxypeptidase A3	-2.933	0.000
**GPIHBP1**	glycosylphosphatidylinositol anchored high density lipoprotein binding protein 1	-2.980	0.000
**DOK6**	docking protein 6	-3.097	0.000
**UNC5A**	unc-5 netrin receptor A	-3.190	0.000
**CLIC5**	chloride intracellular channel 5	-3.200	0.000
**TMEM235**	transmembrane protein 235	-3.290	0.000
**LRRC4**	leucine rich repeat containing 4	-3.395	0.000
**EGFLAM**	EGF like, fibronectin type III and laminin G domains	-3.468	0.000
**THY1**	Thy-1 cell surface antigen	-3.492	0.000
**PTGER3**	prostaglandin E receptor 3	-3.857	0.000
**STC1**	stanniocalcin 1	-3.886	0.000
**ADCYAP1R1**	ADCYAP receptor type I	-4.080	0.000
**RIMKLA**	ribosomal modification protein rimK like family member A	-4.214	0.000
**GABRB2**	gamma-aminobutyric acid type A receptor beta2 subunit	-4.660	0.000
**EGLN3**	egl-9 family hypoxia inducible factor 3	-4.752	0.001
**GPC4**	glypican 4	-4.859	0.000
**PPFIA4**	PTPRF interacting protein alpha 4	-5.087	0.030
**FCMR**	Fc fragment of IgM receptor	-5.652	0.000

Table S3. List of the most disturbed pathways according to the Reactome database.

Pathway identifier	Pathway name
R-HSA-9014843	Interleukin-33 signaling
R-HSA-1566977	Fibronectin matrix formation
R-HSA-111457	Release of apoptotic factors from the mitochondria
R-HSA-205017	NFG and proNGF binds to p75NTR
R-HSA-1253288	Downregulation of ERBB4 signaling
R-HSA-5661231	Metallothioneins bind metals
R-HSA-176974	Unwinding of DNA
R-HSA-111469	SMAC, XIAP-regulated apoptotic response

R-HSA-9020956	Interleukin-27 signaling
R-HSA-8847993	ERBB2 Activates PTK6 Signaling
R-HSA-3595172	Defective CHST3 causes SEDCJD
R-HSA-3595174	Defective CHST14 causes EDS, musculocontractural type
R-HSA-5651801	PCNA-Dependent Long Patch Base Excision Repair
R-HSA-6785631	ERBB2 Regulates Cell Motility
R-HSA-8876725	Protein methylation
R-HSA-5334118	DNA methylation
R-HSA-1266695	Interleukin-7 signaling
R-HSA-8984722	Interleukin-35 Signalling
R-HSA-9012546	Interleukin-18 signaling
R-HSA-2559586	DNA Damage/Telomere Stress Induced Senescence
R-HSA-168256	Immune System
